# Estimating summary measures of health: a structured workbook approach

**DOI:** 10.1186/1478-7954-3-5

**Published:** 2005-05-11

**Authors:** William Flanagan, Jane Boswell-Purdy, Christel Le Petit, Jean-Marie Berthelot

**Affiliations:** 1 Statistics Canada, 24A RH Coats, Ottawa, K1A 0T6, Canada; 2 Public Health Agency of Canada, A. L. 6502D, 130 Colonnade Rd., Ottawa, K1A 0K9, Canada

## Abstract

**Background:**

Summary measures of health that combine mortality and morbidity into a single indicator are being estimated in the Canadian context for approximately 200 diseases and conditions. To manage the large amount of data and calculations for this many diseases, we have developed a structured workbook system with easy to use tools. We expect this system will be attractive to researchers from other countries or regions of Canada who are interested in estimating the health-adjusted life years (HALYs) lost to premature mortality and year-equivalents lost to reduced functioning, as well as population attributable fractions (PAFs) associated with risk factors. This paper describes the workbook system using cancers as an example, and includes the entire system as a free, downloadable package.

**Methods:**

The workbook system was developed in Excel and runs on a personal computer. It is a database system that stores data on population structure, mortality, incidence, distributions of cases entering a multitude of health states, durations of time spent in health states, preference scores that weight for severity, life table estimates of life expectancies, and risk factor prevalence and relative risks. The tools are Excel files with embedded macro programs. The main tool generates workbooks that estimate HALY, one per disease, by copying data from the database into a pre-defined template. Other tools summarize the HALY results across diseases for easy analysis.

**Results:**

The downloadable zip file contains the database files initialized with Canadian data for cancers, the tools, templates and workbooks that estimate PAF and a user guide. The workbooks that estimate HALY are generated from the system at a rate of approximately one minute per disease. The resulting workbooks are self-contained and can be used directly to explore the details of a particular disease. Results can be discounted at different rates through simple parameter modification.

**Conclusion:**

The structured workbook approach offers researchers an efficient, easy to use, and easy to understand set of tools for estimating HALY and PAF summary measures for their country or region of interest.

## Background

Over the past century, advances in public health and population health have dramatically increased life expectancy. Canadians now live longer, but during these added years, they may be affected by disease or chronic conditions. For this reason, indicators used to monitor changes in population health and guide policy decisions need to include how health conditions affect the day-to-day functioning of Canadians over their lifetime.

Summary measures of health that include both mortality and morbidity are being estimated for Canada [1]. Building on prior burden of disease studies by the World Health Organization [2] and Australia [3] that estimated disability-adjusted life years (DALY), the Canadian study will estimate the health-adjusted life years (HALY) lost to premature mortality and reduced functioning for approximately 200 diseases. HALY is computationally identical to DALY; however, it reflects a shift in terminology away from disability towards the broader term health, following recommendations originating from the International Network on Health Expectancy [4].

Estimating summary measures of health requires a wide variety of data including: population counts; incidence and mortality rates; life expectancies; cause-specific and observed survival; distributions, durations, and preference scores across a multitude of health states; and risk factor data to estimate population attributable fractions (PAF). Disaggregating by age group and sex further explodes the quantity of data. To efficiently manage such a large amount of information, we developed a database system, with a set of easy-to-use tools to automatically generate the summary measure estimates.

The main tool generates workbooks, one per disease, that calculate HALY, by importing the data from the database into a generic template. This makes it easy to update the database and quickly regenerate the results. The template is highly structured, which makes the generated workbooks easy to understand and use. There are also tools that summarize the HALY results across diseases for easy analysis. Parameters, such as the rate at which to discount future events, the population of study, and the reference life table, can be specified in the tools to evaluate different scenarios. Furthermore, the generated workbooks are self-contained and can be used directly for specific analysis of a disease. Finally, the tools were built generically to incorporate any number of diseases. Overall, we expect this workbook system will be attractive to other researchers, since it streamlines the process of estimating HALY and PAF, and at the same time provides an organized framework to document the work.

This paper focuses on the system, i.e., tools, database, templates and workbooks, that was developed to estimate the population health impact of cancers in Canada in 2001. Workbooks were generated to estimate health-adjusted life years lost for 26 cancer types (see Table 1) and population attributable fractions for five-related risk factors: alcohol, obesity, lack of fruit and vegetable consumption, physical inactivity, and smoking. The entire application, including a user guide, is available for download in this article's companion zip file.

**Table 1:** Cancer sites by ICD-9 code

**ICD-9**	**Cancer site**
140–149	Oral cancer
150	Esophageal cancer
151	Stomach cancer
153–154,159.0	Colorectal cancer
155	Liver cancer
156	Gall bladder cancer
157	Pancreatic cancer
161	Laryngeal cancer
162	Lung cancer
170–171	Bone and connective tissue cancer
172	Melanoma
173	Non-melanoma skin cancer
174	Breast cancer
180	Cervical cancer
182,179	Uterine cancer
183	Ovarian cancer
185	Prostate cancer
188	Bladder cancer
189	Kidney cancer
191–192	Brain
193	Thyroid cancer
200,202	Non-Hodgkin's lymphoma
201	Hodgkin's disease
203	Multiple myeloma
204–208	Leukemia
All sites between 140–208 not listed above	All other cancers

## Methods

**Figure 1:**
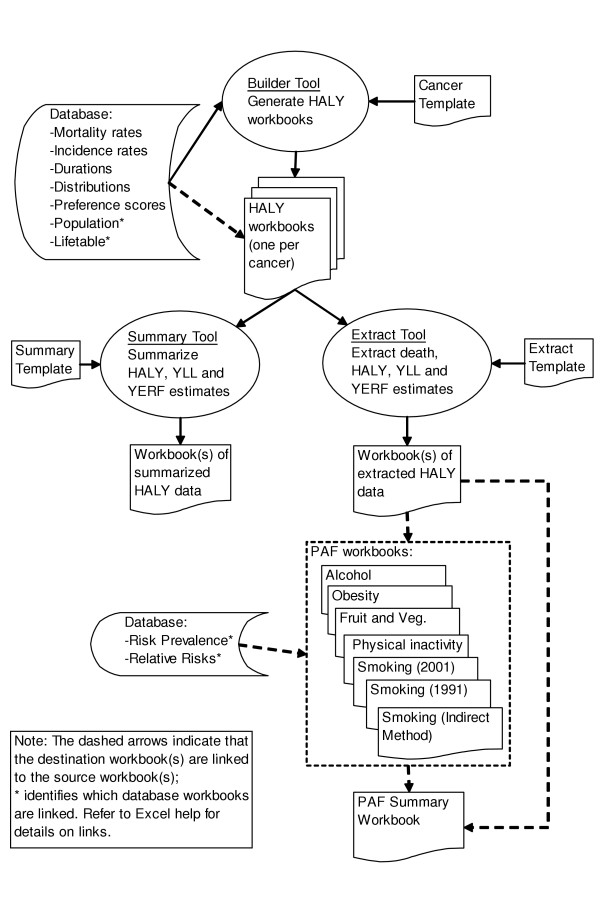
Flowchart of workbook system.

The tools, database, templates, and workbooks that estimate HALY and PAF were developed in Microsoft Excel (version 2002, service pack 2). The interactions between the principal components of the system are shown in the data flow diagram in Figure 1.

### Tools

The tools (identified by ovals in Figure 1) contain imbedded Visual Basic macros that perform three principal functions: generate workbooks to estimate HALY for each of the 26 cancer sites; summarize the HALY results across cancer sites for comparative analysis; and extract HALY results to be attributed to risk factors using population attributable fractions. The tools are called "Builder", "Summary" and "Extract", respectively. Two additional tools discussed below but not shown in Figure 1, are the "Master" and "UpdatePAF" tools. Each tool contains a command button that launches the macro and each has a set of options to control the macro's actions.

### Templates

Each tool uses a template. A template is simply a pre-defined structure that contains formulae and place-holders for data. For instance, the template for the HALY workbooks contains formulae and formatting to calculate the HALY, but it does not contain data. The Builder tool copies the data from the database into the template for a selected disease.

### Links

Some of the data in the generated workbooks are linked to the source files (shown as dashed arrow lines in Figure 1) using a feature of Excel called "links". This means that the data are stored externally to the workbook, but are shown and used in the workbook. The advantage of this approach is that it allows users to quickly change the source of data to easily update the workbook. For instance, the workbooks that estimate HALY can link to any one of the three reference life tables included (or users can create their own life table) to automatically update results. There is complete flexibility in the tool to choose which files to maintain as links. By default, only the population and life table database files are maintained as links. The rest of the data are simply copied from the database to minimize complexity.

### Database

The database is a collection of 17 files organized by the type of data and include: mortality rates; incidence rates; population counts; life expectancy estimates; stage distributions; observed and cause-specific survival; case-fatality estimates; duration and distribution of common cancer health states (diagnosis, treatment, remission, palliative and terminal care); preference scores used to weight for the severity of each health state; utilities that describe the starting health state of the population; risk factor prevalence and relative risk of disease from risk exposure. In addition, three sets of life expectancies have been included in the database: a Canadian multi-cohort life table (2001), a Canadian period life table (1995–1997) and a model life table used by the World Health Organization [5]. The workbooks that estimate HALY link to the multi-cohort life table by default. In general, the database files have been structured by age group, sex, disease, stage, and health state.

To illustrate the workbook system, the database has been populated with Canadian data (or data representative of Canada) for 26 cancer sites and five related risk factors. Cancers were classified by ICD-9 code because of data availability at the time of study. Updating to ICD-10 would not require any change in the structure of the workbook system, only in the data entered into it. The data sources are identified in each of the database files and repeated in the generated workbooks. It is beyond the scope of this paper to discuss the methods used to arrive at this set of data (details available upon request from the authors).

### Workbooks to estimate HALY

#### Algorithm

**Figure 2:**
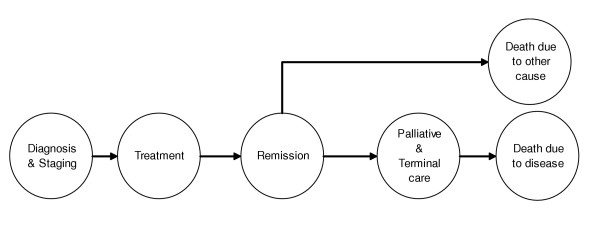
General cancer progression model from diagnosis to death.

Each of the 26 cancer sites were modeled according to a general progression algorithm like the one shown in Figure 2. Although the experience of living with cancer may vary from patient to patient, for practical reasons, we are limited to identifying typical pathways that affect most patients. In this model, cancer patients progress from diagnosis, through a treatment phase to remission and eventually death, from either the cancer or another cause. We model palliative and terminal care phases for cases that die of the cancer.

**Figure 3:**
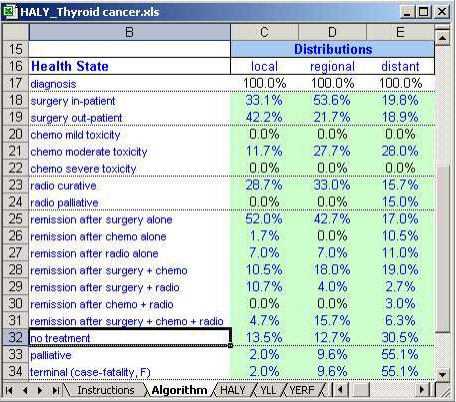
Example of treatment distribution for thyroid cancer.

To improve our cancer model, we divided cancer cases by stage at diagnosis (localized, regional and distant) and disease progression into a set of discrete health states. Treatment options comprise surgery (in-patient and out-patient), chemotherapy (mild, moderate and severe toxicity), hormonal therapy and radiotherapy (curative and palliative). The treatment distributions are entered into the database by type of treatment. For instance, consider the distribution by type of surgery in the workbook for thyroid cancer (Figure 3): 53.2% of patients diagnosed with localized thyroid cancer receive in-patient surgery, 22.2% receive out-patient surgery and the remaining 24.6% do not have surgery. The residual, i.e., the proportion that do not receive surgery in this case, is not explicitly recorded in the database, but is calculated instead. We refer to the period after diagnosis and treatment and prior to the palliative/terminal phase (or death from other cause) as remission.

#### Formulae

The workbooks contain imbedded formulae for calculating the summary measures. HALY is a summary measure that includes both the impact of mortality and morbidity in a single indicator. The mortality component measures the years of life lost due to premature mortality (YLL); the morbidity component quantifies the year equivalents of reduced functioning from living with the disease (YERF). YERF is analogous to years of life lived with disability (YLD) used by the World Health Organization and their collaborators in their burden of disease study; thus, HALY = YLL+YERF is synonymous with DALY = YLL+YLD.

For each cancer site, the HALY, YLL and YERF are estimated by age group and sex according to the following formulae:

HALY_a,s _= YLL_a,s _+ YERF_a,s _   (Eq1)

YLL_a,s _= M_a,s _* L_a,s _   (Eq2)

YERF_a,s _= Σ_g _Σ_e _[l_a,s,g,e_* D_a,s,g,e_* W_g,e_]   (Eq3)

*where a represents the age group, s represents the sex, g represents the stage at diagnosis, e represents the state of progression of the cancer*.

The YLLs are calculated from the number of cases that die from the cancer (M) and the estimated years of remaining life at the age of death (L). The latter is estimated from survival in the general population, by age and sex, and comes from the life table. The death counts are calculated from the mortality rates and the population counts.

The YERFs are calculated by health state and stage at diagnosis. They are estimated from the number of cases entering the health state (I), the duration in the state (D) and the weight for severity of the health state (W). The number of cases entering the health state is derived from the cancer incidence rates, the population counts, the stage distribution and the estimated proportion that experience the health state. For example, the number of women aged 50–54 that receive radiotherapy for cure of localized breast cancer is the product of the number of women in this age group (1,060,244 in Canada in 2001), the incidence rate (229 per 100,000), the estimated proportion that are diagnosed with localized disease (63.6%) and the proportion of these that receive radiotherapy for cure (43.0%), which amounts to 663 cases (these numbers can be found in the breast cancer workbook). The duration of the health state is a direct input parameter, except for the remission/on-going care states, which are calculated as the residual of the overall survival duration less the duration spent in the diagnostic, treatment and palliative/terminal phases.

The weight for severity of the health state is expressed in terms of preference scores (u), as W = 1-u. This assumes full health prior to entering the health state. However, the workbooks allow the population to start in partial health (u_1_) and persist co-morbidly with the cancer state (u_2_). The co-morbidity rule for combining preference scores, u_1 _and u_2_, of two conditions, was defined as:

u_1,2 _= (1- k) * minimum (u_1_, u_2_) + k * (u_1 _* u_2_)

The value of the comorbidity coefficient k was estimated at 0.34, based on a best-fit analysis of Health Utility Index [6] scores for conditions reported in the Canadian Community Health Survey, 2000–01 (CCHS) [7] (details available from the authors). Since we are interested in the reduced functioning *relative *to the initial health state (u_1_), the weight for severity of the cancer health state is given by W = u_1 _- u_1,2_.

#### Discounting

The workbooks include a parameter for discounting the durations of health states that occur at some time T after diagnosis. When a discount rate, r > 0, is specified, the YLL and YERF are estimated according to the modified functional forms:

YLL_a,s _= M_a,s _* (1-e^-rLa,s^)/r   (Eq4)

YERF_a,s _= Σ_g _Σ_e _[l_a,s,g,e_* (1-e^-rDa,s,g,e^)*e^-rTa,s,g,e^]/r * W_g,e_]   (Eq5)

Although the timing and order of treatment, which determines the value of T, may vary from case to case in practice, we assume that treatments occur separately in time and in the following order: diagnosis; surgery; chemotherapy or hormonal therapy; radiotherapy; remission; palliative care; terminal care; and death. The palliative and terminal phases only apply to cases dying of the cancer. The duration preceding them is estimated from the cause-specific survival duration.

We did not include age-weighting in the HALY formulae due to its controversial interpretation [8,9].

#### Structure

**Figure 4:**
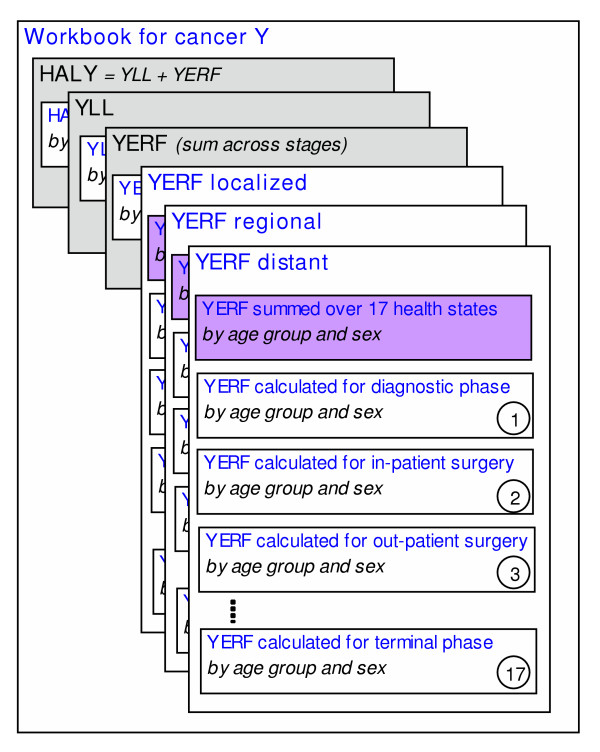
Structure of workbooks for cancer.

Each of the cancer workbooks is structured identically because they originate from the same template. Figure 4 shows a pictorial representation of the cancer workbooks. The HALY and YLL calculations are straightforward implementations of the formulas described above. The total YERF estimate is a sum across the three stages (localized, regional and distant). The worksheets for each stage are structured identically and implement the YERF formulae at the level of the health state. In addition, each of the 17 health state calculations are structured identically, and show the number of new cases entering the health state (I), the duration of the state (D), the time from diagnosis (T), the preference score (u) and the YERF estimate, by age group and sex.

#### Colour scheme

For ease of use, all data elements and parameters that can be modified in the workbooks are identified as green-filled cells. Blue-filled cells are used to highlight labels and violet-filled cells highlight the summary measures.

### Workbooks to estimate PAF

The population attributable fraction (PAF) is an estimate of the proportion of disease in the general population that is due to a particular risk factor. For the study of cancers, workbooks have been developed to estimate the population attributable fraction for five risk factors: alcohol, obesity, lack of fruit and vegetable consumption, physical inactivity, and smoking.

Given the lag time between the exposure to tobacco and the incidence of cancer, and given that the prevalence of smoking has been declining, using current prevalence of smoking will likely produce an underestimation of the population attributable fraction of smoking. In order to quantify this potential bias, we developed three workbooks to estimate the impact of smoking: the first uses current (2001) prevalence of smoking, a second uses prevalence reported in 1991 and the third is an indirect method developed by Peto and Lopez[10].

#### Formulae

For a given risk factor, the PAF is estimated by age group (a), sex (s) and cancer (c) according to the formula:

PAF_a,s,c _= Σ_i _[ Pe_a,s,i _* (RR_a,s,i,c _-1) / (1 + Pe_a,s,i _* (RR_a,s,i,c _-1)) ]   (Eq6)

where Pe is the proportion of the population exposed to the risk factor, RR is the relative risk of developing or dying of cancer due to the exposure, and index i represents the risk category[11]. For instance, the risk categories for obesity are underweight, normal weight, overweight and obese (base on BMI values).

To obtain a more global view of the impact of a risk factor, we produced summary estimates showing the proportion of the total number of cancer deaths, HALY, YLL and YERF attributable to each risk factor by applying the PAF estimates of equation 6 to each of these outcomes. For instance, the impact on deaths for a particular risk factor is given by the formula:

PAFDeaths_s _= [ Σ_c _Σ_a _PAF_a,s,c _*DEATHS_a,s,c_] / [Σ_c _Σ_a _DEATHS_a,s,c _]    (Eq7)

The outcomes are first extracted from the HALY workbooks for a specific discount rate, life table and population choice. They are stored in a separate file and maintained as a link to each of the PAF workbooks. This allows the summary PAF estimates to be easily updated for different parameter choices.

## Results

The tools, database files, templates and workbooks that estimate PAF are all available for download in this article's companion zip file. The workbooks to estimate the HALY need to be generated from the Builder tool after download. After the HALY workbooks have been built for all cancers, the Summary and Extract tools can be used to summarize the HALY results for specific parameter choices, and UpdatePAF tool can be used to update the file links in the PAF workbooks. A higher level tool, the Master tool, has been included to automatically execute these four tasks with the push of one button.

The database is currently populated with cancer data for Canada to illustrate usage, but can be easily adapted for other diseases and updated with data for other countries or regions. To update the database, simply open the database file(s) in Excel and replace the data using standard editing techniques. When adding other diseases, the structure of the database files may be changed to accommodate the number and naming of stages and health states (refer to the user guide for more details).

Here is a brief description of each of the components of the system. More details can be found in the user guide.

### Master Tool

**Figure 5:**
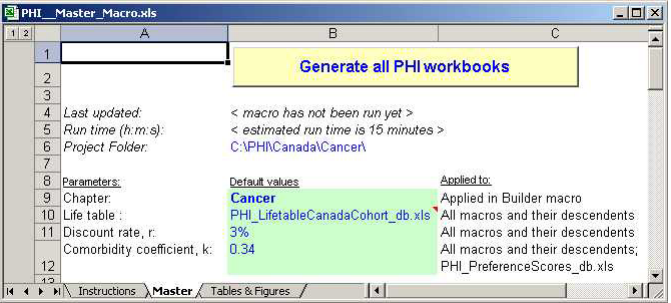
Snapshot of the Master macro tool.

The Master tool runs all of the individual tools – Builder, Summary, Extract, and UpdatePAF – to build a complete system. It allows four parameter choices that are applied throughout the system: the disease chapter for which to run the system (only Cancer is present in this example); the rate at which to discount future events; the comorbidity coefficient to be applied in the rule for combining utilities of two conditions; and the reference life table used to estimate remaining life expectancies. A snapshot of the interface is shown in Figure 5. The *Tables & Figures *worksheet is an advanced feature used to create publication-style tables and figures. It is discussed more thoroughly in the user guide.

### Builder Tool

**Figure 6:**
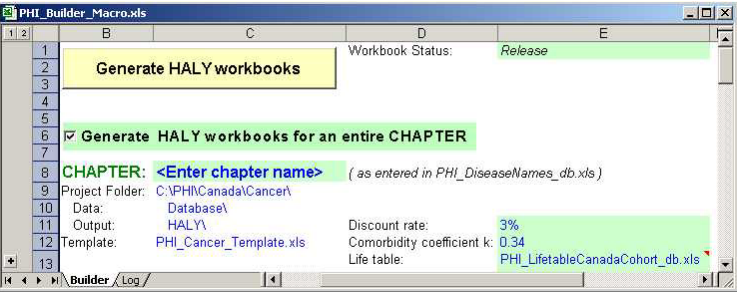
Snapshot of the Builder macro tool.

The purpose of the Builder tool is to generate workbooks that estimate HALY by importing data from the database into a pre-defined template. This allows great flexibility to update the data and recreate the workbooks. The tool, shown in Figure 6, generates one workbook per cancer site. The command button that launches the macro program is at the top of the worksheet and the green-filled cells indicate options that can be specified. The workbooks can be created for the entire cancer chapter or for a specific cancer. Each of the database files required for input to the workbooks is listed by name. Links can be maintained to all of these database files; however it is recommended that links be maintained only for the population and life table files. Other options identify the template file, the location of the database and output folder.

### Summary Tool

**Figure 7:**
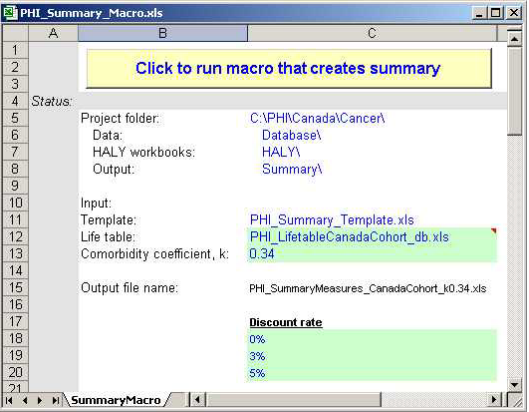
Snapshot of the Summary macro tool.

We designed the Summary tool (Figure 7) to aid in the analysis of the summary measures across all cancer sites. It copies the total HALY, YLL and YERF estimates from each of the cancer workbooks into a single output file. The user can choose parameter values for three discount rates, the life table, and the comorbidity coefficient; these parameters are updated in the HALY workbooks before copying the summary measures to the output file. The parameter choices are automatically reflected in the name of the generated summary file.

### Extract Tool

**Figure 8:**
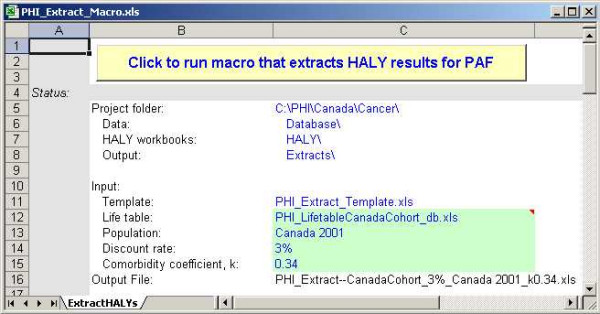
Snapshot of the Extract macro tool.

The Extract tool (Figure 8) was built to facilitate the attribution of summary measures (deaths, HALY, YLL and YERF) to the various risk factors across all cancer sites. The tool simply copies the summary measures, by sex and age group, from each of the cancer workbooks into one file. The data can be extracted under different choices of discount rate, life table, population and comorbidity coefficient. The name of the output file reflects these choices and allows users to create several different extract files for analysis.

### UpdatePAF Tool

The workbooks that estimate population attributable fractions link to the file generated by the Extract tool. The UpdatePAF tool was created to facilitate the update of this link across all eight of these workbooks. The name of the file to be linked is specified in the tool. As with the other tools, UpdatePAF is run automatically by the Master tool.

### Workbooks to estimate HALY

**Figure 9:**
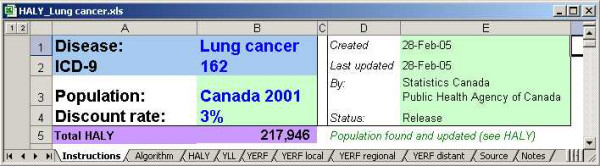
Snapshot of the workbook that estimates HALY for lung cancer.

Once created, the workbooks that estimate HALY can be used as stand-alone workbooks in which parameters or data can be changed. They can also be regenerated at any time with the Builder tool. Figure 9 is a snapshot of the HALY workbook for lung cancer. Each of the workbooks that estimate HALY contains 10 worksheets.

The *Instructions *worksheet offers basic guidance on using the workbooks. It identifies the cancer by name and ICD-9 code. The choice of the reference population and the discount rate are specified in this sheet and applied in all subsequent worksheets. The population counts are displayed in the HALY sheet.

The *Algorithm *worksheet contains the distribution of treatment and remission associated with the cancer, the preference scores for each of the cancer's health states, and utilities that describe the starting health state of the population. It also contains the comorbidity coefficient as a parameter that can be changed. Changing the values here automatically updates the YERF estimates.

The *HALY *worksheet calculates the health-adjusted life years lost as the sum of the YLL and YERF values. The population counts, chosen in the Instructions sheet, are displayed here. The mortality rates are input to the *YLL *worksheet. Population counts and life expectancy estimates are linked data elements used in the calculation of the YLL.

The *YERF *worksheet calculates the total YERF values by summing across stages. The incidence rates for the cancer are found in this sheet. They are combined with the population counts to generate incidence counts, which are then distributed by stage.

The *YERF local, YERF regional, and YERF distant *worksheets calculate the year-equivalents of reduced functioning by health state for each stage, respectively. The stage distribution and the various durations (cause-specific survival, observed survival, duration of treatments, and duration of palliative and terminal care) can be modified. The distribution of treatment and the preference scores are more easily modified in the Algorithm sheet.

The *Sources *worksheet lists every data element used in the workbook, its source and in which worksheet it is found.

The *Notes *worksheet highlights anything exceptional or noteworthy about the cancer.

### Workbooks to estimate PAF

**Figure 10:**
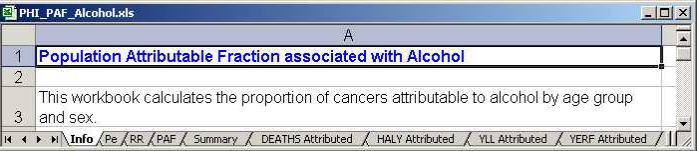
Snapshot of the PAF workbook for alcohol.

There are PAF workbooks for each of the five risk factors. They each contain nine worksheets as shown in Figure 10. The *Info *worksheet provides some basic information related to the calculation of the PAF; the *Pe *worksheet contains risk factor prevalence data, the *RR *worksheet contains the relative risk data for the risk factor and the *PAF *worksheet implements the formulae to estimate PAF by cancer site, age group and sex. In the remaining worksheets, the PAFs are applied to various summary measures, such as HALYs, to estimate how much may be attributable to the risk factor. These results are summarized in the *Summary *worksheet.

The workbook that calculates the PAF associated with smoking by the indirect method includes two additional worksheets with data on the number of lung cancer deaths in a reference population (American Cancer Society, CPS-II, 1984–1988) and in Canada. They are used to estimate the hypothetical proportion that would have to have been exposed to smoking to account for the lung cancer mortality observed in 2001.

### HALY Summary Workbook

**Figure 11:**
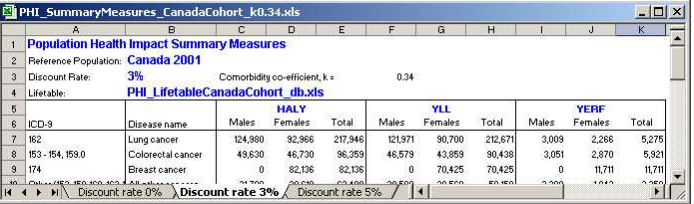
Snapshot of the HALY summary workbook.

Figure 11 is a snapshot of the workbook that summarizes the HALY results. It shows the HALY, YLL and YERF estimates by sex and cancer site, rank ordered by the total HALY. It reports these values for three different discount rates as specified in the Summary tool at the time of creation. The life table and comorbidity coefficient choices are shown and reflected in the file name.

### PAF Summary Workbook

**Figure 12:**
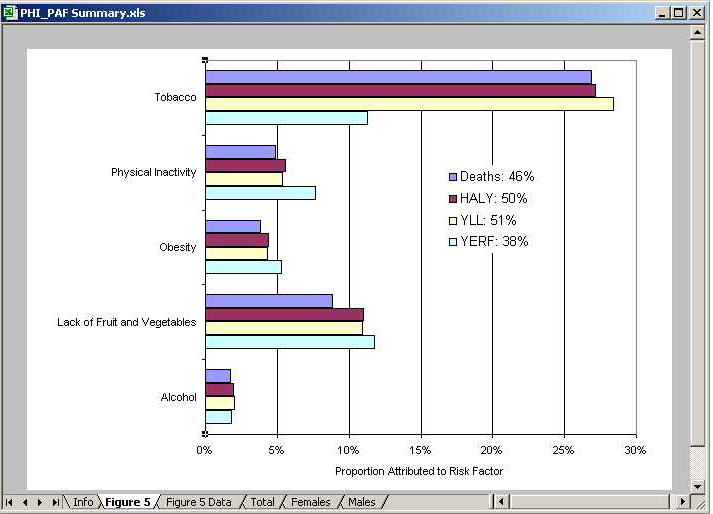
Snapshot of the PAF summary workbook.

The PAF summary workbook (Figure 12) maintains links to each of the individual PAF workbooks for the risk factors and links to the extracted HALY file to obtain the total counts of deaths, HALY, YLL and YERF. It is important that all PAF workbooks link to the same extracted file (the UpdatePAF tool can be use to accomplish this).

## Discussion

The structured approach of the workbook system provides researchers and policy makers with an easy to use and easy to understand tool for estimating HALY and PAF summary measures. Developed for use on personal computers using Excel, it is widely accessible to all levels of researchers. The database can easily be updated with data for other regions or countries and the entire set of results quickly regenerated. Parameter choices in the tools and in the resulting workbooks offer great flexibility to create alternative scenarios. Counterfactuals, used to evaluate the impact of basic health policy interventions, can be created by modifying any of the data elements. For instance, the population attributable fraction associated with obesity could be re-evaluated by reducing the prevalence of obese individuals by an amount that might be achieved by an intervention strategy.

The workbook system has a number of limitations. First, we have not causally linked disease incidence to mortality. Instead, the mortality in 2001 is taken as a proxy for the mortality that would result from the incidence observed in 2001. However, a system that linked incidence to mortality would be more realistic, especially when looking at interventions that reduce incidence. Similarly, survival is not causally linked to treatment in the workbook model. Scenarios that alter treatment patterns would not impact survival time.

A second limitation is that the model of cancer progression does not include the treatment of local or distant recurrence. This means we have not incorporated the weight for severity of these conditions, which would occur during the period labeled remission/on-going care. The preference scores associated with distant cancer are lower than for other stages, so we would expect some underestimation of the HALY by the omission of distant recurrences. This is not expected to have much impact on the ranking of cancers.

Third, the workbook model assumes that cancer treatment follows a fixed sequential order: surgery, chemotherapy, radiotherapy. While the implications for individuals may be extremely important, from a population perspective, and more practically, from the perspective of data availability and model complexity, simplifying assumptions are required. Since the durations of these treatments are relatively short compared to the observed survival of cancer patients, we would expect them to have little impact on the overall outcomes and we would expect their order of occurrence to have even less impact. As a crude sensitivity analysis, we changed the order of occurrence of chemotherapy and radiotherapy in the workbook system, which led to a negligible impact on the estimate of morbidity: overall YERF changed by 0.00025%.

Fourth, clustering of risk factors cannot be easily modeled in the workbooks. This means that the proportion of cancer attributable to one risk factor may also be attributed to another risk factor, even though the two risk factors collectively contribute to the cancer. For instance, alcohol and smoking may be clustered risk factors with respect to death from laryngeal cancer, which may explain why our estimate of the total proportion of laryngeal cancer deaths exceeds 100%. In general, we expect we have overestimated the population attributable fractions across all cancers.

Finally, the data have been obtained at levels of disaggregation to represent the heterogeneity of the cancer population. However, in the case of the calculated duration of remission, it has not been sufficient to avoid logical inconsistencies. We found that in older age groups, it was possible to generate negative durations of remission, because the observed survival of people in this age group was less than the duration spent in diagnostic, treatment and terminal phases. The input data could be refined to avoid this, but as a rare occurrence with small impact, we opted to check for negative durations and set them to zero when they occur. This is done automatically by the imbedded formulae and requires no intervention by the user.

These limitations can be overcome through more advanced modeling techniques, such as microsimulation modeling. The Population Health Model, a continuous time, competing risk microsimulation model developed at Statistics Canada, is being adapted to implement all of the functionality of these workbooks. Our experience with both types of modeling suggests that it is not necessarily more difficult to develop the microsimulation model, although it is often considered less transparent. The benefit of developing both models is that the workbooks provide a benchmark against which the microsimulation results can be compared. Of course, we expect small differences, not only from random noise introduced by the stochastic nature of the microsimulation model, but also because it avoids the limitations outlined above.

The workbook system presented here focused on cancers. However, it was developed more generally, so that it can produce workbooks for other chronic diseases or injuries, once the data have been assembled. The main criterion is that the disease(s) can be decomposed into a series of health states from diagnosis to death. The number of health states is virtually unlimited and can include disease progression and sequelae, and the diseases can be staged at diagnosis or not. By changing a few labels in the database files to reflect health state names, an entire new system of HALY workbooks can be generated (refer to user guide for detailed steps). The underlying macro code has been built with this generalizability in mind.

As with any generalized system, exceptions may arise that do not fit within its structured framework. As more diseases are studied in the Canadian study, the workbook structure will be modified or expanded to address any exceptions that arise. This may take the form of minor modifications to the current structure, a separate structure to accommodate multiple disease exceptions that fall into a common framework, or a series of ad-hoc, stand-alone workbooks for unique exceptions. We expect that most diseases will fit within the structure described here. Future releases of the workbook system will be made through the Public Health Agency of Canada's website[1].

## Conclusion

The structured workbook approach offers researchers an efficient, easy to use, and easy to understand set of tools for estimating HALY and PAF summary measures for their country or region of interest. The estimation of summary measures for cancers presented here highlights the functionality of the system; however, the tool is easily expanded to other diseases. The workbooks are transparent in their calculations, but are limited in their ability to model the impact of clustered risk factors and competing risks of disease. These limitations can be overcome by more advanced modeling techniques such as microsimulation.

## Competing Interests

The author(s) declare that they have no competing interests.

## Authors' contributions

WF participated in the conceptualization and testing of the workbook system, designed and developed the system, and drafted the manuscript. CLP and JBP participated in the conceptualization and testing of the workbook system. JMB provided the principal guidance in the conceptualization of the workbook system. All authors have read and approved the manuscript.

## Supplementary Material

Additional File 1 PHI Workbook System. This 2.6 Mb zip file for download contains the Excel files that comprise the workbook system. The system estimates summary measures of health and is initialized with cancer data for Canada. When unzipped, the workbook system will occupy approximately 6 Mb of additional disk space and a folder structure will be created under *C:\PHI*, where you will find the user guide. Click here for file
