# Supplementary material for: Estimating summary measures of health: a structured workbook approach
Source: Popul Health Metr. 2005 May 11;3:5. doi: 10.1186/1478-7954-3-5 (PMC1156945; doi:10.1186/1478-7954-3-5)
Supplement: Additional File 1 — PHI Workbook System. This 2.6 Mb zip file for download contains the Excel files that comprise the workbook system. The system estimates summary measures of health and is initialized with cancer data for Canada. When unzipped, the workbook system will occupy approximately 6 Mb of additional disk space and a folder structure will be created under C:\PHI, where you will find the user guide. [file 1478-7954-3-5-S1.zip › PHI/userguide.doc]

**A user guide to the PHI Workbook System**

***A database system with tools to estimate summary measures of health***

William Flanagan, Statistics Canada

Table of Contents

Table of Figures	1

Intended Use	2

Data Requirements	2

System Requirements	2

Installation	3

Folder Structure	3

Overview	5

Getting Started	7

*The Master Tool*	7

*The Builder Tool	9*

*The Extract Tool	12*

*UpdatePAF Tool	12*

*Tables and Figures tools	13*

Frequently Asked Questions (FAQs)	13

*If the Master tool can do everything, why do I need the other macro tools ?	13*

*How do I work with the individual HALY workbooks ?	13*

*How do I estimate population attributable fractions associated with risk factors ?	13*

*How do I estimate the impact of risk factors ?	14*

*Do I have to enable macros when opening a tool ?	14*

*Should I edit links when opening a workbook ?	14*

*How do I customize the database for my country or region ?	14*

*If I rename the Canada folder to reflect my country, will the system still work ?	14*

*How do I add other diseases to the workbook system?	14*

*How do I udpate from ICD-9 to ICD-10 ?	16*

Future Releases	16

Contact Information	16

**Table of** Figures

Figure 1: PHI workspace	3

Figure 2: Database folder	4

Figure 3: PAF folder	4

Figure 4: Data flow diagram of PHI workbook system	6

Figure 5: Master tool	8

Figure 6: Builder tool.	9

Figure 7: Summary tool	11

Figure 8: Extract tool	12

Figure 9: UpdatePAF tool	12

**Intended Use**

This tool will be of interest to researchers who would like to embark on a burden of disease study for their country or region. The objective of the tool is to estimate summary measures of health; principally, health-adjusted life years (HALY) lost to premature mortality and reduced functioning for a variety of diseases. HALY is analogous to the DALY (disability-adjusted life years) used in burden of disease studies by the World Health Organization and Australia; it reflects a shift in terminology from disability towards functional health, with the aim of using utilities that measure the functional health associated with disease states.

A secondary outcome of the workbook system is population attributable fractions (PAF), used to evaluate the proportion of disease outcomes (such as deaths or HALYs) that are attributable to different risk factors.

For a more thorough discussion, please refer to the companion article "Estimating summary measures of health: a structured workbook approach" published in Population Health Metrics.

This work is part of a larger research program, the Population Health Impact of Disease in Canada (PHI). More information is available at the following website:

<http://www.phac-aspc.gc.ca/phi-isp/index.html>

Future releases of the PHI workbook system will be available through this website or by contacting the authors.

**Data Requirements**

To estimate these summary measures requires a great deal of data. This version of the workbook system contains cancer related data estimated in the Canadian setting. Countries or regions with limited or partial data may still be able to benefit from the workbook system by collapsing certain dimensions or by finding representative data from other regions.

The minimum data elements needed to estimate HALYs are: population counts; mortality rates; incidence rates; cause-specific and observed survival; case-fatality; and preference scores (or other utility measures). Canadian preference scores for cancers, derived using the Classification and Measurement System of Functional Health (CLAMES) [developed by Statistics Canada], are included in the workbook system. Preference scores for other diseases are being derived and will be made available upon request, through the website noted above. Utility measures may also be available from other burden of disease studies, such as those used in The Burden of Disease and Injury in Australia [Mathers et. al.].

Elements that can be simplified include the age group dimension, the sex dimension, the number of health states and the stage dimension. Use only those aspects for which there is data and ignore the others. For instance, if stage data were not available for cancers, then data could be entered as if one stage and the other two stages could be blanked. Similarly, if detailed treatment data was not available, they could be left blank in the database with no loss of functionality.

To estimate population attributable fractions, one needs relative risks and prevalence for the applicable risk factors, by disease.

**System Requirements**

You must have Microsoft Excel 2002 with service pack 2, or higher, to run the macros included in this package. To download and unzip, you will require approximately 8Mb of disk space. An additional 25Mb of disk space will be required to generate the workbooks. Multiple copies of the PHI workbook system can be installed.

**Installation**

Download the 2.6Mb PHI_Workbook_March2005.zip file to your local drive (C:) and unzip it. This user guide is found in folder *C:\PHI.*

**Folder Structure**

When the file is unzipped to the root of your C: drive it creates a folder structure, *C:\PHI\Canada\Cancer* as shown in Figure 1.

Five macro files (tools) and three template files are found in the root of the Cancer folder, as shown in Figure 1. The remaining files are found in subfolders Database, Extracts, HALY, PAF, Summary and Tables. Figures 2 and 3 show the contents of the Database and PAF folders. The other folders - Extracts, HALY, Summary and Tables - are populated with files generated by the macro tools.

The macro of primary interest to users will be *PHI__Master_Macro.xls*; it runs all other macros to completely build the workbook system.

Figure 1: PHI workspace

Figure 2: Database folder

Figure 3: PAF folder

**Overview**

The Population Health Impact of Disease in Canada (PHI) workbook system for cancers comprises (prior to running macros):

- five macro tools
- three template files
- 17 database files
- eight predefined workbooks for estimating the population attributable fraction (PAF) by risk factor
- a subfolder (*Tables*) of macros, templates and other files used to generate data lists in pivot table format, for assisting with analysis and creating report tables

After the macros are run to "initialize" the system, HALY, PAF and summary workbooks are generated for a particular set of parameter choices:

- 26 HALY workbooks (one per cancer) that estimate the health-adjusted life years (HALY) lost due to premature mortality and year-equivalents of reduced functioning
- one workbook of summary results (HALY, YLL, YERF) copied from the HALY workbooks
- one workbook of summary results (HALY, YLL, YERF and death counts) extracted from the HALY workbooks and formatted for use with the PAF workbooks
- an update of the values in the eight PAF workbooks based on links to the extracted HALY data
- other workbooks that summarize the HALY results in pivot table format (in *Tables*)

The workbook system is initialized by running the Master macro, *PHI__Master_Macro.xls*. It runs each of the individual macros - Builder, Extract, Summary, UpdatePAF and Tables - that produce the HALY, PAF, and summary workbooks. The interactions between the core tools Builder, Extract and Summary, and the workbooks they create are shown in the data flow diagram in Figure 4.

Figure 4: Data flow diagram of PHI workbook system

*Understanding the Data Flow Diagram:*

The direction of the arrows indicates the flow of data in and out of the processes. For instance, the process called *Builder tool* uses data from several database files as well as the cancer template, and it generates workbooks (one per cancer) as the result. These are represented by the solid arrow lines entering and leaving the process. Dashed arrow lines indicate that source files are maintained as links. For instance, the HALY workbooks generated by the Builder tool maintain links to three of the database files: the preference score, population and life table files. (See Excel help for more details on links.)

**Getting Started**

After downloading and unzipping the workbook system onto your C drive, you will need to decide whether you want to use the cancer data for Canada (as included in the download package), or whether you want to replace some or all of it with data for another country or region. You may also duplicate the cancer example to model other diseases.

To explain how the workbook system functions, the discussion here will focus on the example of cancers. Thus, we take as a starting point that the database is complete for the cancer chapter, and that our goal is to produce workbooks that estimate HALY for 26 cancer sites, PAF for five cancer-related risk factors and several tables that summarize the results across all cancers. All workbooks can easily be generated using only one tool, the Master tool. However, we will discuss all of the tools - Master, Builder, Extract, Summary, UpdatePAF and Tables- to offer a better understanding.

***The Master Tool***

The Master tool is the recommended starting point, since it ensures the integrity and completeness of the workbook system and requires minimal intervention from the user. The tool's interface is illustrated in Figure 5.

The Master tool, *PHI__Master_Macro.xls*, runs:

- the Builder tool to generate HALY workbooks,
- the Summary tool to summarize HALY results for all cancers,
- the Extract tool to summarize HALY results for input to the PAF workbooks,
- the UpdatePAF tool to update links in PAF workbooks to the extracted HALY results, and
- the Tables tools that summarize the HALY data in pivot table format to facilitate analysis and creation of table and figures.

*Steps:*

1. Open *PHI__Master_Macro.xls*
2. Click on the worksheet called *Master*
3. Specify parameter choices (defaults supplied)
4. Run the macro by clicking on the command button at the top of the window.

*Notes:*

- Initializing the system means generating the HALY, PAF and various summary workbooks from template and database files. N.B. The data must be entered into the database before running the macros.
- Set the parameters (chapter, discount rate, comorbidity coefficient, and life table) as desired in cells B9:B12 (this example only contains the Cancer chapter in the database).
- Run the initialization macro when you will not be using your computer for other tasks (it takes 15-30 minutes on most computers).
- The initialization macro opens, displays and automatically runs each of the individual macros listed in cells B16:B22, in the order listed.
- When this macro is finished, the *Master* workbook reappears (permanently) and the *Last updated* date and time are reset in cell B4.

*Tables & Figures worksheet*

This is an advanced feature of the workbook system because it requires knowledge of Excel pivot tables. The Tables sub-component of the workbook system is described later in this guide. More details are can also be found in the *Instructions* and *Table & Figures* worksheets of *PHI__Master_Macro.xls.*

Figure 5: Master tool

## The Builder Tool

The Builder tool, *PHI_Builder_Macro.xls,* generates HALY workbooks for an individual cancer or an entire chapter. This is run automatically by the Master macro (described above); however, it can be opened and run independently. Figure 6 illustrates the tool and its default settings. Each of its main features is described subsequently.

Figure 6: Builder tool.

*Generate HALY workbooks*

The macro is run by clicking on the yellow-filled command button *Generate HALY workbooks*. It uses the parameters and file information specified. It takes approximately one minute to generate one workbook. While the macro is running, you will notice other Excel files opening and closing. Please wait until the macro is finished, indicated by a message in cell C5, before continuing to use Excel.

*Generate HALY workbooks for an entire chapter (or for a specific disease)*

You have the choice to generate workbooks for an entire chapter or for a particular disease. This is achieved by clicking on the checkbox next to *Generate HALY workbooks for an entire CHAPTER*. Enter the name of the chapter or the name of the specific disease in cell C8. The database contains only cancer data at this time. The chapter name or disease name must match those found in *PHI_DiseaseNames_db.xls.*

For example, to generate the entire cancer chapter, ensure that the checkbox is activated and enter Cancer at the prompt (cell C8). To generate only the lung cancer workbook, enter Lung cancer at the prompt.

As the macro runs, it displays the name of the current disease being processed in cell B4, and also makes an entry in the log sheet when it has been completed.

*Parameters*

The discount rate (r), comorbidity coefficient (k), and life table are parameter choices specified in cells E11, E12, and E13, respectively. These choices are applied to (and saved in) the generated workbooks.

*Project Folder and subfolders*

A formula is used in cell C9 to automatically identify the location of the workbook system. We recommend that you do not manually enter this field. The name of the database folder and output folder are also specified here (cells C10 and C11).

*Cancer Template*

A default template called *PHI_Cancer_Template.xls* is supplied (identified in cell C12). This is a pre-defined template that determines the structure of the HALY workbooks.

*Advanced: Database files*

The database files used by this macro are listed in cells C15 to C27. It is unlikely that a user would change these settings, but it is possible. They correspond to the file names found in the Database subfolder.

*Advanced: Links*

The macro can copy the data from the database files as values or as linked data to the source files. For each database file there is a flag (in column A) that indicates whether to break the link ("Break"), i.e., copy the data as values, or to maintain the link ("Maintain").

Linked data appear as values in the workbook but the data source is actually another Excel file. With linked data, changes to the source file are automatically updated in the workbooks that link to them.

The default settings maintain links to the preference score, life table and population database files. Maintaining links to the preference score database file is required when planning to experiment with different values of the comorbidity coefficient parameter after the system is fully built.

***The Summary Tool***

Another way to review the results in the HALY workbooks is to generate a summary report using the Summary tool *PHI_Summary_Macro.xls* (Figure 7).

As with the Builder tool, the folder locations and name of the template file have been defaulted and should not be manually changed.

Operationally, the summary tool opens each HALY workbook, applies the parameter choices temporarily (i.e., does not save them to file), and copies the summary information (HALY, YLL and YERF) to the template file, by sex. When all HALY workbooks are processed, it saves the summary file, named according to the parameter choices made. This way multiple summary files can be created, reflecting different parameter choices, and they are all stored in the Summary folder.

Note that the Master tool automatically runs this tool, updates the comorbidity coefficient and the life table, but does not update the discount rates (defaulted to 0, 3 and 5%).

Figure 7: Summary tool

## The Extract Tool

The Extract tool, *PHI_Extract_macro.xls*, opens each of the HALY workbooks, applies the parameter choices, and copies the deaths, HALY, YLL and YERF into a summary file. The summary file contains entries for each cancer site by age group and sex. The name of the summary file is based on the parameter choices made and is saved in the subfolder *Extracts\*. Figure 8 illustrates the interface for setting the parameters and running the macro.

Figure 8: Extract tool

## UpdatePAF Tool

The workbooks that estimate population attributable fractions link to the file generated by the Extract tool. The UpdatePAF tool (Figure 9) was created to facilitate the update of this link across all eight of these workbooks. The name of the file to be linked is specified in the tool. As with the other tools, UpdatePAF is run automatically by the Master tool.

Figure 9: UpdatePAF tool

## Tables and Figures tools

The Tables sub-component of the PHI workbook system (found in folder *Tables*) was added to assist in analysis of the results and in the production of publication-style tables and figures. This section is advanced in that it requires knowledge of how to create and use Excel pivot tables (refer to Excel documentation).

The Tables sub-component consists of three subgroups, generically identified as A, B and C, which each consist of a macro, template and pre-defined pivot table(s). A set of publication-style table and figure shells (mock-ups) have been designed and are found in *PHI_Tables_and_Figures.xls* (section D).

Like the other summary macros described earlier, the Tables’ macros copy data from the HALY workbooks and pastes them into a list format that can be used with the pivot table feature of Excel.

When the Master tool is run to build the workbook system, it also runs these Tables’ macros and generates these lists. The *Tables & Figures* worksheet of the Master tool facilitates using the pre-defined pivot tables, but users may find it easier to simply create their own pivot tables directly from the lists. Furthermore, the results are not automatically copied from the pivot tables to the publication-style tables.

More information can be found in the *Instructions* and *Tables & Figures* worksheets of *PHI__Master_Macro.xls.*

# Frequently Asked Questions (FAQs)

## If the Master tool can do everything, why do I need the other macro tools ?

The Master tool is a convenient way to run all of the other tools that build the system starting from the templates and database. It runs them in the proper order and ensures the data links are up-to-date.

The other tools can be opened and run independently. This may be of particular interest for those adding a new disease, if they wish to generate a single HALY workbook to verify that it works.

Another use of the summary tools is to generate different result files for different parameter choices, along the lines of sensitivity analysis. However, it may still be easier to perform the same sensitivity analysis by installing multiple versions of the workbook system, each with its own parameter assumptions.

## How do I work with the individual HALY workbooks ?

You may wish to open one or more of the HALY workbooks in Excel and familiarize yourself with the calculations and the data. You can modify data and parameters directly in the workbook to experiment with the impact on the estimate of HALY. Refer to the *Instructions* worksheet of these workbooks for more information. Alternatively, the Summary and Table tools can be used to summarize and analyze the results from the HALY workbooks.

## How do I estimate population attributable fractions associated with risk factors ?

The population attributable fraction (PAF) is an estimate of the proportion of the total risk of a disease in the general population that is due to a particular risk factor. For the study of cancers, workbooks have been developed to estimate the population attributable fraction for five risk factors: alcohol, obesity, lack of fruit and vegetable consumption, physical inactivity, and smoking. In general, these workbooks contain two types of data to estimate PAF: the relative risk of disease due to exposure to the risk factor; and the prevalence of the risk factors, or exposure rates, in the population. The data can be updated for other regions or changed to experiment with counterfactuals. In addition, the PAF is applied to various summary measures of health, such as estimated number of deaths, HALY, YLL and YERF, to estimate the impact of the risk factors on each, by cancer site, sex and age group.

## How do I estimate the impact of risk factors ?

The eight pre-defined PAF workbooks calculate the population attributable fractions for each of the cancer-related risk factors (alcohol, lack of fruit and vegetables, obesity, physical inactivity, tobacco).

To attribute the number of deaths, HALY, YLL and YERF to each of the risk factors, the PAFs are applied to these outcomes (by cancer, sex and age group). Evidently, these outcomes must first be generated using the Builder tool and put into a format designed for use with these PAF workbooks. The latter is accomplished with the Extract tool (described below).

## Do I have to enable macros when opening a tool ?

Yes. Whenever you open an Excel workbook that contains macros you will be prompted to disable or enable macros. Enabling macros allows the PHI Workbook System to function. The system has been virus scanned prior to release.

## Should I edit links when opening a workbook ?

When you open an Excel workbook that contains links, you may be asked to edit the links. In general, it is not necessary to edit the links, since they should be up-to-date. For instance, when the Master tool is run, it ensures that all links are up-to-date. If you are in doubt, then you may edit the links, either from this prompt, or at any time from the menu choices Edit \ Links. Refer to Excel Help for the mechanics of updating links.

Also refer to UpdatePAF for more options on updating the links to the PAF workbooks.

## How do I customize the database for my country or region ?

If you have data for another country or region and would like to enter it into the system, open the appropriate database file(s) in Excel and use standard Excel editing techniques to replace the existing data. No automatic data validation is performed. See Data Requirements section for more details.

## If I rename the Canada folder to reflect my country, will the system still work ?

Yes. The workbooks contain formulae that automatically identify the location of the opened workbook. In fact, the subfolder *Cancer* can also be renamed, if you wish to model another disease (see section *How do I add other diseases to the workbook system?)*.

Renaming of the lower level subfolders (Database, etc) is not recommended as it would require modification of the entries in the macros. Furthermore, the macro files and the other subfolders must remain at the same level, as shown in Figure 1.

## How do I add other diseases to the workbook system?

The example described in this user guide focused on the implementation of cancers; however the tool is easily adapted for other diseases. By changing a few labels in the database files and entering the country-specific data for those diseases, an entire new set of HALY workbooks can be generated. The underlying macro code has been built with this generalizability in mind.

The main criterion to fit within the workbook structure is that the disease(s) can be decomposed into a serious of health states from diagnosis to death. Health states can include treatment options, disease progression, sequelae, remission, and terminal states. In addition to health states, diseases can be split by stage at diagnosis, where applicable, as demonstrated for cancers. Diseases which cannot be decomposed in this manner may require ad-hoc development of new structures; however, we expect that most diseases and injuries will fit into this framework.

Here is a list of the steps required to implement a new disease chapter.

Step 1: Create a new copy of the workbook system from the zip file.

a. Rename the subdirectory from "Cancer" to the name of the new chapter.
b. Blank the cancer data from the database.

Step 2: Add the disease names to the database:

a. Open the PHI_DiseaseNames_db.xls
b. Add an entry for each disease, with the appropriate ICD9 (or ICD10) code and chapter name.

Step 3: Modify the names of the health states and assign sequence numbers

a. Open the PHI_PreferenceScores_db.xls, PHI_HSDistributions_db.xls, and PHI_HSDurations_db.xls.
b. Rename the health states as required. Names must be consistent across all three db files. Columns may be added or deleted.
c. The set of health states must always include a diagnosis state identified with sequence number 00, a terminal state identified with sequence 99, and at least one remission state (sequence numbers 80-89).
d. Sequence numbers are assigned in the preference score database.
e. Sequence numbers must be assigned to all health states. This is used to automatically calculate the time from diagnosis to the beginning of the health state for the purposes of discounting.
f. For mutually exclusive health states with the same timing, assign the same sequence number.
g. The health states do not have to be ordered by sequence number in the database file.
h. The distribution to the diagnosis state is always 100% (required for internal consistency of database and programs). It means that 100% of the incidence cases distributed to the stage experience an “at diagnosis” phase.

Step 4: Modify or add new stages.

a. Open PHI_StageDistribution_db.xls, PHI_SurvivalCauseSpecific_db.xls, PHI_SurvivalObserved_db.xls.
b. Delete, rename or add new worksheets for each stage required. If stage is not applicable, use "StageNA" as the worksheet name, and use it as the value for the stage where required elsewhere in the database (100% of the stage distribution is assigned to “StageNA”).
c. Currently, the workbooks can handle a maximum of four stages per disease.
d. Ensure that the stage distribution adds to 100% across the applicable stages of a disease.

Step 5: Data entry.

a. Open each database file, add the disease name in the appropriate place, and stage if applicable, and enter your data.

Step 6: Add risk factors.

a. To estimate population attributable fractions (PAF) for other diseases, risk factors must be identified and their prevalence and relative risk of developing disease(s) obtained. The existing database files and the workbooks that calculate PAF will help guide you to create your own custom workbooks.

## How do I update from ICD-9 to ICD-10 ?

The choice of disease classification is made independently of the workbook system. That decision determines how you will retrieve your data to put into the workbooks, but the operation of the workbook system does not change as a result.

For clarity of labeling in the workbook system, it is recommended that the label in the PHI_DiseaseNames_db.xls database file be identified appropriately prior to running the system. Simply type in the appropriate label that describes the classification system used in cell C1 (in our example, it is written as ICD-9). Labels in all other workbooks are automatically updated upon system initialization.

# Future Releases

The Population Health Impact of Disease in Canada research program will continue to model other diseases and expand the PHI workbook system. Future releases of the software will be available at our website:

<http://www.phac-aspc.gc.ca/phi-isp/index.html>

# Contact Information

You may contact us by email at:

hamg@statcan.ca

phi-isp@phac-aspc.gc.ca
